# Cutaneous Tuberculosis among Patients Presenting to Dermatology Outpatient Department of a Tertiary Care Centre: A Descriptive Cross-sectional Study

**DOI:** 10.31729/jnma.7930

**Published:** 2023-01-31

**Authors:** Niraj Parajuli, Anupama Karki, Ashesh Dhungana

**Affiliations:** 1 Department of Dermatology & Venereology, National Academy of Medical Sciences, Mahaboudha, Kathmandu, Nepal; 2 Department of Chest Medicine, National Academy of Medical Sciences, Mahaboudha, Kathmandu, Nepal

**Keywords:** *cutaneous*, *extrapulmonary tuberculosis*, *tuberculid*

## Abstract

**Introduction:**

Cutaneous tuberculosis is an uncommon form of extrapulmonary tuberculosis. It can present in various morphological presentations leading to a late diagnosis in many cases. It is mainly associated with significant scarring and morbidity. It is classified as paucibaciUary or multibaciUary depending on the bacillary load. Similarly, it can be acquired through either an endogenous or an exogenous source. The mainstay of treatment is anti-tubercular medications. The objective of the study was to find out the prevalence of cutaneous tuberculosis among patients presenting to the dermatology outpatient department of a tertiary care centre.

**Methods:**

A descriptive cross-sectional study was done among the patient presenting to the Outpatient Department of Dermatology and Venerology in a tertiary care centre where all patients data from medical records were taken from April 2016 to March 2021 after taking ethical approval from the Institutional Review Committee (Reference number: 503/2078/79). Demographic details of the patients including age, sex, site and duration of the lesion were recorded. Convenience sampling was done. Point estimate and 95% Confidence Interval were calculated.

**Results:**

Among 1,30,924 cases, 40 (0.03%) (0.02-0.04, 95% Confidence Interval) cutaneous tuberculosis was seen.

**Conclusions:**

The prevalence of cutaneous tuberculosis was similar to the studies done in similar settings.

## INTRODUCTION

Cutaneous tuberculosis (CTB) is a chronic infection caused by *Mycobacterium tuberculosis, Mycobacterium bovis* and in certain instances by Bacille Calmette-Guerin (BCG) vaccine. CTB is a relatively uncommon presentation of tuberculosis (TB) compromising only about a 1% of all extrapulmonary manifestations.^1^

There is a lack of data on cutaneous TB in Nepal. The cutaneous form can present with different morphologies depending on the bacterial load, host immunity, infection source, and transmission route.^2^ It can be further classified as multibacillary or paucibacillary and exogenous or endogenous according to the source of infection.^3^ The presentation can mimic many other skin diseases that might lead to misdiagnosis and hence delay in treatment. CTB is known as the great imitator. Most relies on the presence of typical granulomas in histopathological examination.^3,4^

The objective of the study was to find out the prevalence of cutaneous tuberculosis among patients presenting to the dermatology outpatient department of a tertiary care centre.

## METHODS

A descriptive cross-sectional study was done among the patient presenting to the outpatients Department of Dermatology & Venereology, National Academy of Medical Sciences, Bir Hospital, Kathmandu, Nepal where data from medical records were taken from April 2016 to March 2021 after taking ethical approval from the Institutional Review Committee (Reference number: 503/2078/79). Demographic details of the patients including age, sex, site and duration of the lesion were recorded. Convenience sampling was done. The sample size was calculated using the following formula:


$$\begin{array}{ll} \text{n} & ={\text{Z}}^{2}\times \frac{\text{p}\times \text{q}}{{\text{e}}^{\text{2}}}\\ & =1.96^{2}\times \frac{0.50\times 0.50}{0.01^{2}}\\ & =9604 \end{array}$$

Where,

n = minimum required sample size
Z = 1.96 at 95% Confidence Interval (CI)
p = prevalence is taken as 50% for maximum sample size calculation
q = 1-p
e = margin of error, 1%

The calculated sample size was 9604. Decuple the sample size minimum required sample was 96040. All patients with a final diagnosis of cutaneous tuberculosis and histopathological features suggestive of CTB will be included in the study. Similarly, all the detailed histopathology reports and Mantoux test results were also included. Any patients without complete details were excluded from the study.

Data collected were entered and analyzed using IBM SPSS Statistics version 25.0. Point estimate and 95% Confidence Interval were calculated.

## RESULTS

**Figure 1:**
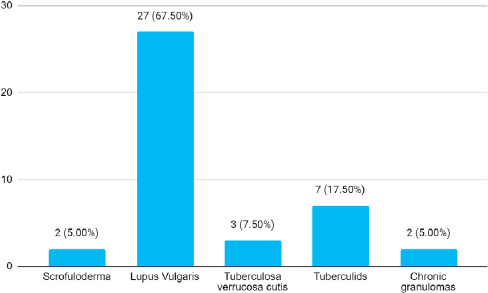
Distribution of different cutaneous tuberculosis forms over a 5-year period (n= 40).

Among 1,30,924 cases, 40 (0.03%) (0.02-0.04, 95% CI) cutaneous tuberculosis was diagnosed. The mean age group was 36.47 years. The prevalence was higher in females 25 (62.50%) compared to males 15 (37.50%) with a ratio of 1.6:1. The disease duration range from 10 days up to 26 years. The most common type of cutaneous TB was Lupus vulgaris present in 27 (67.50%) patients. One (2.5%) patient was noted with two different forms of CTB together namely Tuberculosa verrucosa cutis (TVC) and scrofuloderma (Figure 1).

**Figure 2:**
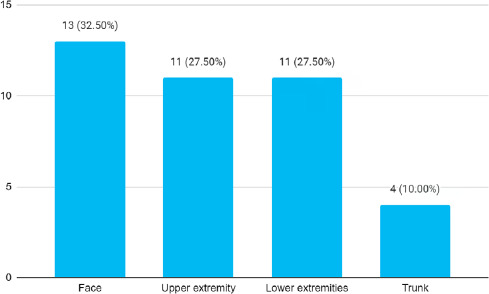
Distribution according to the site of involvement (n= 40).

All of the tuberculids presented with multiple lesions. Plaque was the most common presenting skin lesions in 33 (82.50%) patients. Among these, ulceration was present in only 4 (10%) patients. However, the ulcer was the presenting sign in only 2 (5.00%) patients. Facial involvement was the most common site 13 (32.50%) (Figure 2).

All patients with erythema induratum had nodules in the lower legs. All of the patients had strongly positive Mantoux tests which range from 10 mm induration to up to 25 mm induration. Past history of tuberculosis was elicited in only 2 (5%) patients with lupus vulgaris and a family history of pulmonary TB was present in 3 (7.5%) patients of CTB. Histologically, all of the specimens showed features of chronic granulomatous infiltration. The presence of acid-fast bacilli was noted only in a single case of scrofuloderma. There was no active pulmonary tuberculosis noted in all cases of CTB.

## DISCUSSION

CTB is among the rarest presentation of TB and our study found a prevalence of 0.03% of CTB. There are various morphological types of CTB described. The presentation of which depends on the immune status of the patient, bacterial load, route and bacillary load.^2^ It can further be classified as paucibacillary and multibacillary types. Tuberculous chancre, scrofuloderma, orofacial tuberculosis, acute military TB and gumma consist of high bacillary load whereas TVC, lupus vulgaris and tuberculids have a low bacillary load.^3^ These consists of papulonecrotic tuberculids, lichen scrofulosorum, erythema induratum of Bazin and nodular tuberculids.

Mantoux test or PPD test is a classic example of a delayed-type hypersensitivity reaction. Although the interpretation is difficult, this test is commonly used during a suspicion of CTB.^5^ The test's sensitivity ranges from 33%-96% whereas the specificity is around 60% with a 10 mm cut-off. The typical histology is the presence of granulomas with multinucleated giant cells with the presence or absence of caseation. Other useful tests include culture of the bacilli, polymerase chain reaction, genotyping and restriction fragment length polymorphism. However, these tests are expensive and not easily available. More so, the PCR test has a low sensitivity in cutaneous TB.^3^ Antitubercular medications are the mainstay of treatment.

Nepal is endemic for TB, it reported that 68,000 people developed TB in 2019.^6^ The proportion of EP tuberculosis was around 9194 but the prevalence of CTB is missing even from the national data. CTB can present with significant scarring with associated morbidity and rarely mortality. About 0.1%, which is a small proportion compared to the huge burden of pulmonary tuberculosis in Nepal. This study was a data retrieval of CTB diagnosed in a tertiary centre referral centre in Nepal that will help us understand the probable picture of CTB in Nepal.

CTB prevalence in our study was similar to other studies done globally but less than others in the South Asian countries.^4,7^ Studies from India, have shown a greater number prevalence as compared to our study.^8-10^ Multiple published papers have mentioned a similar number of patients as in our study with exception of studies from Pakistan and Ethiopia.^11,12-16^

Contrary to our study and Brazilian study, other studies from Nepal and India have shown a male predominance.^11,4,9^ The most common clinical variant of CTB was lupus vulgaris in our study which was similar to other studies from Nepal, Pakistan and India.^4,9,14^ However, studies from Tunisia, India, Brazil and Ethiopia have found scrofuloderma as the most common form.^8,11,15,17^ This shows that systemic TB involvement was more common in these countries. Erythema induratum was the most common form of tuberculids in our study which presented with multiple tender nodules over bilateral lower legs. The face was the most common site for CTB, predominantly lupus vulgaris.

This study was conducted only in a single institute with a limited sample. A larger study of a nationwide sample should be conducted to better understand the exact prevalence.

## CONCLUSIONS

The prevalence of cutaneous tuberculosis was similar to studies done in similar settings. Cutaneous forms of tuberculosis are uncommon and there is a lack of adequate data. An effort should be made by the national TB programs to estimate the nation wide prevalence and recommend the best possible treatment of CTB in our scenario.
